# Analysis of performance structures of activities of daily living affecting the sleep using social network analysis

**DOI:** 10.1002/alz.088776

**Published:** 2025-01-09

**Authors:** Sungmin Son, Mose Hwang, Aeri Shim, Hyeyoung Lee, Kyuho Jeong, Yeonjoo Nam, Taehui Kim

**Affiliations:** ^1^ Yonsei university, Wonju‐si, Gangwon‐do Korea, Republic of (South); ^2^ Yonsei University, Wonju‐si, Gangwon‐do Korea, Republic of (South)

## Abstract

**Background:**

Sleep directly affects daily life, and lack of sleep affects cognitive function and mental health. So, this study analyzed the performance structures of daily activities affecting sleep using social network analysis.

**Methods:**

The subjects were 313 people over 50 years old. The performance structures of daily activities were analyzed by social network analysis, and network characteristics, centrality, and cohesion structures were analyzed. Daily activities were analyzed by categorizing into pre‐sleep daytime activities, sleep preparation activities, and post‐sleep daytime activities. Data collection utilized a structured survey. Subjects were allowed to describe up to five activities to affect sleep. Intensity was assessed using a 5‐point scale.

**Results:**

The analysis of network characteristics showed that the number of activities performed in the daytime was the most performed. However, during sleep preparation, due to the decrease in the number of activities, the density increased, and the values of degree and eigenvector centrality showed high. The results of network centrality showed that, before sleep, bathing/showering, physical activities, and job performance and maintenance influence sleep. During sleep preparation, turning off lights was the most affecting activity on sleep. After sleep, eating and swallowing was the most affected activity by the results of sleep. The results of cohesion analysis showed that, before sleep, job performance and maintenance formed the structures associated with socializing with others. Eating and swallowing, and physical activities formed the structures associated with the self‐management activities performed. In preparing for sleep, turning off lights formed the structure associated with organizing the environment for sleep. After sleep, eating and swallowing formed the structures associated with the basic daily activities related to self‐management.

**Conclusions:**

This study analyzed the interaction between sleep and daily activities. Based on these, key interventions for optimizing sleep include reinforcing bathing/showering and physical activities, as well as regulating job performance and maintenance before sleep. Organizing the environmental factors affecting to sleep and encouraging basic daily activities post‐sleep are needed. The findings can be used as important information to enhance sleep‐related activities of daily living.

